# Replication Requires Psychological Rather than Statistical Hypotheses: The Case of Eye Movements Enhancing Word Recollection

**DOI:** 10.3389/fpsyg.2016.02023

**Published:** 2016-12-26

**Authors:** R. Hans Phaf

**Affiliations:** ^1^Amsterdam Brain and Cognition Center, University of AmsterdamAmsterdam, Netherlands; ^2^Department of Psychology, Brain and Cognition Group, University of AmsterdamAmsterdam, Netherlands

**Keywords:** confirmatory research, replication, eye movements, SIRE, EMDR, hypothesis-guided research

## Abstract

Can an experiment be replicated in a mechanical fashion without considering the processes underlying the initial results? Here I will consider a non-replication of Saccade Induced Retrieval Enhancement (SIRE) and argue that it results from focusing on statistical instead of on substantive process hypotheses. Particularly the theoretical integration of SIRE with Eye-Movement Desensitization and Reprocessing (EMDR) therapy, provides clues about when the memory enhancement should occur. A relatively large memory enhancement effect in participants with a consistent (i.e., extreme right or left) handedness should be observed, (a) when explicitly instructed to retrieve and imagine the memories during the eye manipulation, and (b) for emotionally negative material. A finer theoretical analysis may thus well explain the contrast between the original SIRE studies and the non-replication. Also the findings from preregistered confirmatory research (i.e., focusing solely on statistical hypotheses) should be considered preliminary, representing shifts on a gradual scale of evidence, and awaiting interpretation in terms of theoretical hypotheses. Stronger, but still not definitive, conclusions can better be postponed until after multi-study meta-analyses with theoretically motivated moderator variables have been performed.

“There are many hypotheses in science which are wrong. That’s perfectly all right; they’re the aperture to finding out what’s right. Science is a self-correcting process. To be accepted, new ideas must survive the most rigorous standards of evidence and scrutiny.”

Carl Sagan (1990, Cosmos: A Personal Voyage, Heaven, and Hell [Episode 4] 33 min 20 s)

Many published research findings are undoubtedly ‘false’ (i.e., incorrect), also when bias or questionable research practices (QRPs) are completely absent (Ioannidis, 2005). An even larger number cannot be reproduced in replication research (Open Science Collaboration, 2015). To remedy this undesirable situation, Wagenmakers et al. (2012) advocated that researchers should preregister their studies and specify their statistical tests in advance (i.e., “confirmatory” research). Only for this type of purely confirmatory research would the common statistical tests be valid. Although these authors note that there is nothing wrong with their other type of research (i.e., “exploratory” research), as long as this is explicitly acknowledged, they do associate exploratory research with “wonky” statistics. Confirmatory research procedures may to some extent be able to reduce bias and QRPs, but may still not be able of turning a majority of false published findings into a minority (“…the pure gold standard is unattainable.” p. 700, Ioannidis, 2005).

Tests serve as a means for making short-term decisions about statistical hypotheses, which in turn can each correspond to an infinitely large number of different substantive process hypotheses. Such decisions may be highly fallible due to the occurrence not only of bias and QRPs but also of inadvertent technical and data processing errors. Even if the effect exists, moreover, it is expected to not become significant a number of times (i.e., the power never equals one), and the false negative rate may even be higher than the false positive rate (cf, Fiedler et al., 2012). No conclusion about non-significant effects can be drawn either way, and probably a preference for the initial hypothesis should be kept (cf, Dienes, 2014). Even if it becomes significant moreover, the finding may frequently be false (i.e., without any bias or QRPs; Ioannidis, 2005). Stroebe et al. (2012) were equally pessimistic about the self-corrective power in psychology and other fields of science (e.g., even physics), but limited their analysis to scientific fraud cases for which they noted that short-term self-correction by replication and peer review did not seem to work. Confirmatory research practices would thus only filter out a percentage of false findings (and miss many “true” findings), but the remainder probably requires a longer and more gradual process of theoretical considerations and comparisons to other findings.

I would argue that self-correction in science, also of bona-fide but false claims, mostly does not result from active discussions or confirmatory research, but from a more passive quasi-Darwinian selection of ideas and hypotheses working on longer time scales (e.g., generations of scientists). Evolutionary development probably represents the most powerful optimization process available, and may well also apply to science (cf, Holland, 1975; Dawkins, 1986). The above fallibility of statistical tests severely limits their contribution to the quasi-evolutionary selection process. More often, false hypotheses are ignored in the long run (i.e., become “extinct”), whereas hypotheses that are more consistently supported by the evidence and fit in ongoing discussions have higher chances of survival and reproduction. The single-experiment support or rejection of a statistical hypothesis in the purely confirmatory view can better be replaced by a multi-experiment weighing of psychological hypotheses, which can be represented as different levels of a theoretically motivated moderator variable in a meta-analysis (cf, Ioannidis, 2005). Even after a meta-analysis, one cannot be completely sure that one’s decisions about particular hypotheses are “true.” This is also not too dissimilar from the optimization process performed by evolution. Scientific development can also get stuck in local optima, not being able to reach even fitter solutions.

Replication studies conforming to confirmatory standards may still be useful instruments, as long as they are sufficiently theoretically informed. In a purely mechanical view on replication, however, researchers try to reproduce statistical outcomes of tasks rather than predictions of well-specified theories (cf, Klein, 2014). These replicators run the risk of neglecting important moderators that may also not have been recognized explicitly in the initial, to-be-replicated study. Such a hidden variable may inadvertently have been set to different values in the original and the replication studies, which can even lead to opposing outcomes. If in this case falsification is erroneously concluded from non-replication, this may hamper the development of science rather than fostering it. It is certainly true that the hidden moderators invoked by non-replicated researchers may sometimes appear trivial (e.g., testing in cubicles), and unrelated to theory (for this critique, see Yong, 2012; Klein, 2014), but I will discuss an example of a non-replication where these variables could have been derived *a priori* from prominent theories in the field. The non-replication by Matzke et al. (2015) of Saccade-Induced Retrieval Enhancement (SIRE; e.g., Lyle et al., 2008) seems to suffer from such a theoretical neglect. Determining whether the initial result or the non-replication is “false” does not seem possible by statistical tests alone, but also requires consideration of the underlying process hypotheses and their associated hidden variables.

## Eye Movements, Memory, and Emotion

Two main fields of eye movement (EM) research, sharing an interest on memory processing after short periods of EMs, were linked by Matzke et al. (2015). SIRE investigates the enhancement of predominantly emotionally neutral memories after executing EMs (e.g., Lyle et al., 2008). Eye Movement Desensitization and Reprocessing therapy (EMDR; Shapiro, 1989) deals with the emotional processing of traumatic and anxious memories due to EMs (e.g., Armstrong and Vaughan, 1996; Lee and Cuijpers, 2013). The original SIRE studies did not refer to EMDR, although the eye manipulation, involving a 30 s period of EMs at a 1 s pace, was very similar to the therapeutic procedure. The to-be-retrieved material, moreover, did not consist of traumatic memories, as in EMDR therapy, but of low-to-medium frequency, largely affectively neutral, words.

In contrast to the growing confidence in the effectiveness of EMDR (e.g., van den Hout and Engelhard, 2012; Lee and Cuijpers, 2013), the evidence for SIRE has suffered from the non-replication by Matzke et al. (2015). These authors joined in an adversarial-collaboration replication study as proponents or skeptics of SIRE and could not reproduce the memory enhancement obtained by Lyle et al. (2008). Bayesian statistics revealed that the observed data were 15 times more likely under H_0_ (i.e., no difference) than under H_1_ (i.e., a difference in memory performance between eye manipulation conditions). The proponents in this study were not convinced by this single failure to replicate, but the skeptics even raised the possibility of bias and QRPs on the side of the SIRE research community to explain the initial finding. This conclusion does not seem warranted due to the high rate of false positives (cf, Ioannidis, 2005) and false negatives (cf, Fiedler et al., 2012) even in the absence of bias and QRPs. In addition, it disregards theoretical reasons for the discrepancy.

Only one account for SIRE was considered in the non-replication (i.e., the hemispheric interaction hypothesis; Lyle et al., 2008, 2012), which had previously been dismissed by the proponents in the adversarial collaboration (Samara et al., 2011). Other influential accounts, primarily for EMDR, such as the working memory account (Andrade et al., 1997) or the orienting response account (Armstrong and Vaughan, 1996; Stickgold, 2002), as well as the newer top-down attentional control account from the SIRE domain (Edlin and Lyle, 2013; Lyle and Edlin, 2015) were completely ignored. Elsewhere (Phaf, submitted), I have identified crucial hidden variables based on the linking of theoretical accounts for SIRE and EMDR that may well explain the contrast between the original SIRE findings and the non-replication.

Two variables suggest themselves from the application of EMDR accounts to SIRE. Neither the retrieval, and re-imagining, during EMs, nor the emotionality of the memories were deemed important in SIRE research. Matzke et al. (2015) even explicitly suppressed the former influences by including a recency buffer at the end of the study list, and moreover strictly selected for affectively neutral words. Because Lyle et al. (2008, 2012) had the EMs performed immediately after study, some recently presented words may still have been active during the EMs. Also the absence of selection for neutrality here meant that there could have been an unknown proportion of negative words in the list. In a non-preregistered (i.e., exploratory, in the statistical classification of Wagenmakers et al., 2012) study an explicit retrieval instruction during the eye manipulation, and the strict selection of negative material, has strongly amplified memory enhancement, far exceeding the effect sizes commonly reported for SIRE (Phaf, submitted). However, also this experiment cannot yield conclusive evidence concerning the crucial hidden factors, but should be followed up by further research that explicitly compares instructions to re-imagine with attempts to suppress such retrieval. The control over, or lack of, or attempts to actively suppress, memory re-activation during EMs could then serve as a moderator variable in meta-analyses of SIRE. The valence of the studied material, as well as the absence of control over valence, could be another moderator variable. To corroborate the present hypotheses, the largest effect sizes should be obtained with memory (re-activation) during EMs and for negative material. Instead of getting bogged down in a statistical impasse, such, probably exploratory, research would eventually advance our understanding of SIRE and may even help to improve EMDR.

## Replication Requires Theory

Statistical testing is not a goal in itself in Psychology, but the development of theory is. The confirmatory type of research proposed by Wagenmakers et al. (2012) tries to validate the statistical tests, but does not necessarily provide meaning to the results. Without a theoretical specification of the hypotheses even significant findings can mean anything, and their application (e.g., in case of practical interventions) may remain “magical” (as has been argued for EMDR, McNally, 1999). The non-replication of Matzke et al. (2015) provides an example of not sufficiently addressing theory. Process hypotheses could have been derived here not only from EMDR but also from other potential sources (e.g., visual attention, working memory). This a-theoretical stance is fostered by an over-reliance on statistical tests. The practice of only describing test statistics but not actual results (e.g., means and measures of variance) in results sections of research papers (e.g., many studies had to be excluded for this reason from the meta-analysis of Phaf et al., 2014) further illustrates the frequent prioritization of mechanical statistical testing over theoretical analysis. The emphasis in these papers should shift from establishing that “something is there” to estimating and explaining what exactly is happening in the results.

Too often statistical testing acts as a stop criterion, which consists of the simple decision rule that an effect is there if it is significant and not there if it is non-significant, taken to indicate that no further theoretical analysis is needed. The non-replication of Matzke et al. (2015) may have reached this stop criterion even earlier, not after the tests were performed but in the initial stage when the tests were planned and preregistered. Even more theoretical work is required, however, after non-significance than after significance. If one considers a theoretical hypothesis to be refuted by non-significance, a superior alternative should always be formulated according to modern philosophy of science (e.g., Lakatos, 1970). There can be no hypothesis abandonment without hypothesis replacement. The utilization of the stop criterion distinguishes mechanical replication attempts of statistical hypotheses from theoretically informed replication attempts of substantive hypotheses. Some researchers even use it as a tool for relieving them from the burden of having to delve into a largely confusing abundance of prior findings and hypotheses. The stop criterion frequently results in what Ioannidis (2005) calls the Proteus phenomenon that squarely contradicting, but both significant, sets of results are published shortly after another, sometimes even in the same journal, without referring to the other. The opposing findings are not necessarily caused by bias or QRPs in one of the studies, but may simply reflect the majority of bona-fide significant findings being false, as Ioannidis argues. Another unfortunate consequence of this criterion is that the same research is often repeated over and over again (i.e., “the wheel is reinvented”), sometimes with slight modifications or (e.g., neuro-imaging) additions, while the researchers remain unaware of previous work. Due to the frequent application of the statistical stop criterion for theoretical analysis, psychology often does not seem to learn from its own research.

In terms of substantive hypotheses, classical null hypothesis statistical testing performs a kind of inverse, rather than direct, falsification. Instead of trying to falsify a concrete hypothesis, one tries to establish evidence against being nothing there. After rejecting the null hypothesis, one claims that this rejection supports one’s proposed hypothesis, which may take any form other than the null. The H_1_ thus extends to an infinite range of theories, and could better be renamed H_∞_ to recognize the theoretical indifference of this hypothesis. This contrasts sharply with the dominant falsification practice in for instance physics. Here a non-trivial hypothesis is disconfirmed when the values predicted by theory fall outside the uncertainty interval around the observed results (e.g., Taylor, 1982). This approach compares predicted and actual results and concludes to non-falsification in the absence rather than presence of a difference. The physics approach to data analysis also entails more attention for measurement accuracy than in psychology. Non-significance in classical null hypothesis statistical testing more often indicates a lack of measurement accuracy than an absence of difference, however small it may be (cf, Cohen, 1990). Physical theories are undoubtedly among the most numerical and highly developed in the whole of science, and therefore probably better suited to this approach than psychological theories. The rigid application of statistical hypothesis-testing, however, seems to have aggravated the neglect of theory in psychology.

A single-experiment just cannot serve to decide conclusively whether a claim is false or not (cf, Hauer, 2004; Ioannidis, 2005). It merely adds weight, proportional to the accuracy of its measurements, to one or the other position. A publication of a new effect should be considered suggestive, but certainly not definitive “proof” (cf, Phaf et al., 2014). In the words of Medawar (1991): “In the outcome science is not a collection of facts or of unquestionable generalizations, but a logically connected network of hypotheses which represent our current opinion about what the real world is like.” (p. 98) Scientific exaggeration is often required by funding agencies for research marketing purposes (also called “valorization” at Dutch universities), but may induce QRPs and even fraud. Scientific prudence and modesty seem better ways to reach a durable development of science. Confirmatory researchers may inadvertently add to this exaggeration, because they are inclined to think of science as collecting conclusive, sometimes even “proven,” facts, whereas history has shown it to consist of ongoing discussions with continuous weight shifts between alternative hypotheses (cf, Lakatos, 1970).

If null hypothesis statistical testing detracts from psychological hypotheses and even induces a false sense of certainty, why not abandon null hypothesis statistical testing altogether (cf, Cumming, 2014)? The reporting of only effect sizes and confidence intervals (CIs) may actually reduce publication bias, because the latter is based more often on significance levels than on effect sizes (cf, Simonsohn et al., 2014). In addition, these estimation statistics are more informative, because they, similar to physics, focus on what the effect is rather than on what it is not. CIs should be used as an indication of measurement accuracy rather than for making decisions on whether some unspecified “effect” is there or not (e.g., contains zero; see Gardner and Altman, 1986). The latter decisions are highly fallible (a majority is probably “false,” see Ioannidis, 2005; Fiedler et al., 2012), and we need other, more theoretical, arguments to determine the level of support for a hypothesis provided by a set of results. Stronger, but still not infallible, conclusions can better be postponed until after meta-analyses on the proposed hypotheses have been performed (Schmidt, 1996). These meta-analyses have the additional advantage of identifying publication bias and being able to correct for it with the Trim-and-Fill method (Duval and Tweedie, 2000), or possibly with the p-curve method (Simonsohn et al., 2014). Only when effect size and the extent of publication bias can be judged in a meta-analysis, one can have more confidence in a finding.

The primary aim of this comment is to juxtapose the statistically oriented approach and a more theoretically oriented approach. The statistical approach of Wagenmakers et al. (2012) entails a two-way classification in either exploratory or hypothesis-confirmatory research. The latter type can only have a binary outcome with respect to the decision being made, the hypothesis is either confirmed or not. To arrive at such an outcome, a replication attempt must rely on the original research having uncovered and made explicit all relevant processes (i.e., an exhaustive theoretical analysis). All other types of research fall in the exploratory category, even when they further develop the theory starting from quite specific hypotheses. Merely confirming preregistered hypotheses has, however, never yielded new hypotheses, whereas unexpected findings stimulating further investigations do have that capacity and may even be the royal road to scientific innovation (e.g., Lehrer, 2009). Calling it undirected exploratory research, moreover, also does not do justice to the gradual progress-by-adjustment type of research (cf, Lakatos, 1970). The latter type of research is often guided by well-specified and concrete process hypotheses, which may be far superior above merely expecting a difference. Although I think we should try to move away from null hypothesis statistical testing (cf, Cumming, 2014), in the meantime a statistical approach to experimental psychology should become more theoretically oriented and include a third category: hypothesis-guided research.

## Author Contributions

The author confirms being the sole contributor of this work and approved it for publication.

## Conflict of Interest Statement

The authors declare that the research was conducted in the absence of any commercial or financial relationships that could be construed as a potential conflict of interest.

## References

[B1] Andrade J. Kavanagh D. Baddeley A. (1997). Eye-movements and visual imagery: A working memory approach to the treatment of post-traumatic stress disorder. *Br. J. Clin. Psychol.* 36 209–223. 10.1111/j.2044-8260.1997.tb01408.x 9167862

[B2] Armstrong M. S. Vaughan K. (1996). An orienting response model of eye movement desensitization. *J. Behav. Ther. Exp. Psychiatry* 27 21–32. 10.1016/0005-7916(95)00056-9 8814518

[B3] Cohen J. (1990). Things I have learned (so far). *Am. Psychol.* 45 1304–1312. 10.1037/0003-066X.45.12.1304

[B4] Cumming G. (2014). The new statistics: why and how. *Psychol. Sci.* 25 7–29. 10.1177/0956797613504966 24220629

[B5] Dawkins R. (1986). *The Blind Watchmaker.* New York, NY: Norton.

[B6] Dienes Z. (2014). Using Bayes to get the most out of non-significant results. *Front. Psychol.* 5:781 10.3389/fpsyg.2014.00781 PMC4114196 25120503

[B7] Duval S. Tweedie R. (2000). Trim and fill: a simple funnel-plot–based method of testing and adjusting for publication bias in meta-analysis. *Biometrics* 56 455–463. 10.1111/j.0006-341X.2000.00455.x 10877304

[B8] Edlin J. M. Lyle K. B. (2013). The effect of repetitive saccade execution on the attention network test: enhancing executive function with a flick of the eyes. *Brain Cogn.* 81 345–351. 10.1016/j.bandc.2012.12.006 23485024

[B9] Fiedler K. Kutzner F. Krueger J. I. (2012). The long way from α-error control to validity proper problems with a short-sighted false-positive debate. *Perspect. Psychol. Sci.* 7 661–669. 10.1177/1745691612462587 26168128

[B10] Gardner M. J. Altman D. G. (1986). Confidence intervals rather than P values: estimation rather than hypothesis testing. *Br. Med. J.* 292 746–750. 10.1136/bmj.292.6522.746 3082422 PMC1339793

[B11] Hauer E. (2004). The harm done by tests of significance. *Accid. Anal. Prev.* 36 495–500. 10.1016/S0001-4575(03)00036-8 15003595

[B12] Holland J. H. (1975). *Adaptation in Natural and Artificial Systems.* Ann Arbor, MI: The University of Michigan Press.

[B13] Ioannidis J. P. A. (2005). Why most published research findings are false. *PLoS Med.* 2:e124 10.1371/journal.pmed.0020124 PMC1182327 16060722

[B14] Klein S. B. (2014). What can recent replication failures tell us about the theoretical commitments of psychology? *Theory Psychol.* 24 326–338. 10.1177/0959354314529616

[B15] Lakatos I. (1970). “Falsification and the methodology of scientific research programmes,” in *Criticism and the Growth of Knowledge*, eds Lakatos I. Musgrave A. (Cambridge: Cambridge University Press).

[B16] Lee C. W. Cuijpers P. (2013). A meta-analysis of the contribution of eye movements in processing emotional memories. *J. Behav. Ther. Exp. Psychiatry* 44 231–239. 10.1016/j.jbtep.2012.11.001 23266601

[B17] Lehrer J. (2009). *Accept Defeat: The Neuroscience of Screwing Up.* Available at: http://www.wired.com/2009/12/fail_accept_defeat/2/ [accessed April 14, 2015]

[B18] Lyle K. B. Edlin J. M. (2015). Why does saccade execution increase episodic memory retrieval? A test of the top-down attentional control hypothesis. *Memory* 23 187–202. 10.1080/09658211.2013.877487 24499320

[B19] Lyle K. B. Hanaver-Torrez S. D. Hackländer R. P. Edlin J. M. (2012). Consistency of handedness, regardless of direction, predicts baseline memory accuracy and potential for memory enhancement. *J. Exp. Psychol.* 38 187–193. 10.1037/a0024831 21843021

[B20] Lyle K. B. Logan J. M. Roediger H. L. (2008). Eye movements enhance memory for individuals who are strongly right-handed and harm it for individuals who are not. *Psychonomic Bull. Rev.* 15 515–520. 10.3758/PBR.15.3.515 18567248

[B21] Matzke D. Nieuwenhuis S. van Rijn H. Slagter H. A. van der Molen M. W. Wagenmakers E. J. (2015). The effect of horizontal eye movements on free recall: a preregistered adversarial collaboration. *J. Exp. Psychol. Gen.* 144 e1–e15. 10.1037/xge0000038 25621378

[B22] McNally R. J. (1999). EMDR and mesmerism: a comparative historical analysis. *J. Anxiety Disord.* 13 225–236. 10.1016/S0887-6185(98)00049-8 10225510

[B23] Medawar P. (1991). *The Threat and the Glory: Reflections on Science and Scientists.* Oxford: Oxford University Press.

[B24] Open Science Collaboration (2015). Estimating the reproducibility of psychological science. *Science* 349 aac4716 10.1126/science.aac4716 26315443

[B25] Phaf R. H. Mohr S. E. Rotteveel M. Wicherts J. M. (2014). Approach, avoidance, and affect: a meta-analysis of approach-avoidance tendencies in manual reaction time tasks. *Front. Psychol.* 5:378 10.3389/fpsyg.2014.00378 PMC4021119 24847292

[B26] Samara Z. Elzinga B. M. Slagter H. A. Nieuwenhuis S. (2011). Do horizontal saccadic eye movements increase interhemispheric coherence? Investigation of a hypothesized neural mechanism underlying EMDR. *Front. Psychiatry* 2:4 10.3389/fpsyt.2011.00004 PMC3089996 21556274

[B27] Schmidt F. L. (1996). Statistical significance testing and cumulative knowledge in psychology: implications for training of researchers. *Psychol. Methods* 1 115–129. 10.1037/1082-989X.1.2.115

[B28] Shapiro F. (1989). Eye movement desensitization: a new treatment for post-traumatic stress disorder. *J. Behav. Ther. Exp. Psychiatry* 20 211–217. 10.1016/0005-7916(89)90025-6 2576656

[B29] Simonsohn U. Nelson L. D. Simmons J. P. (2014). P-curve and effect size correcting for publication bias using only significant results. *Perspect. Psychol. Sci.* 9 666–681. 10.1177/1745691614553988 26186117

[B30] Stickgold R. (2002). EMDR: a putative neurobiological mechanism of action. *J. Clin. Psychol.* 58 61–75. 10.1002/jclp.1129 11748597

[B31] Stroebe W. Postmes T. Spears R. (2012). Scientific misconduct and the myth of self-correction in science. *Perspect. Psychol. Sci.* 7 670–688. 10.1177/1745691612460687 26168129

[B32] Taylor J. R. (1982). *An Introduction to Error Analysis: The Study of Uncertainties in Physical Measurements.* Sausalito, CA: University Science Books.

[B33] van den Hout M. A. Engelhard I. M. (2012). How does EMDR work? *J. Exp. Psychopathol.* 3 724–738. 10.5127/jep.028212

[B34] Wagenmakers E. J. Wetzels R. Borsboom D. van der Maas H. L. Kievit R. A. (2012). An agenda for purely confirmatory research. *Perspect. Psychol. Sci.* 7 632–638. 10.1177/1745691612463078 26168122

[B35] Yong E. (2012). In the wake of high profile controversies, psychologists are facing up to problems with replication. *Nature* 483 298–300. 10.1038/485298a

